# Nomogram for predicting severe abdominal pain after initial conventional transarterial chemoembolisation for hepatocellular carcinoma: a retrospective study

**DOI:** 10.1038/s41598-023-49509-z

**Published:** 2023-12-16

**Authors:** Huixia Qin, Xuhua Xiao, Houxiang Ya, Jinhai Li, Fugang Liang, Haiqing Jin, Lianghuan Liao, Yaohua Li, Jiahui Qin, Jue Yu, Jing Gu, Chunmei Zhou, Ming Jin, Ying Miao, Shuqun Li

**Affiliations:** 1 Department of Interventional Radiology Center, Affiliated Hospital of Guilin Medical University, Guilin, China; 2 Department of Gastroenterology, Affiliated Hospital of Guilin Medical University, Guilin, China; 3 Department of Liver and Gall Surgery, The Third Affiliated Hospital of Wenzhou Medical University, Zhejiang, China; 4 Department of Hepatobiliary and Pancreatic Surgery, Affiliated Hospital of Guilin Medical University, Guilin, China; 5 Department of Vascular Intervention, Affiliated Hospital of Guilin Medical University, Guilin, China

**Keywords:** Liver cancer, Pain

## Abstract

Transarterial chemoembolisation (TACE) is a standard therapy for hepatocellular carcinoma (HCC). However, adverse events, including abdominal pain, are common. This study aimed to investigate and verify the feasibility of a nomogram model to predict severe abdominal pain after first conventional TACE (cTACE) among patients with HCC. Patients with HCC treated with cTACE between October 28, 2019, and August 5, 2022, at a single centre were enrolled (n = 216). Patients were divided into training and validation cohorts (ratio, 7:3). A visual analogue scale score between 7 and 10 was considered severe abdominal pain. A total of 127 (58.8%) patients complained of severe abdominal pain after first cTACE treatment. The nomogram considered age and tumour number and size. The nomogram demonstrated good discrimination, with a C-index of 0.749 (95% confidence interval [CI], 0.617, 0.881). Further, the C-index in the validation cohort reached 0.728 (95% CI 0.592, 0.864). The calibration curves showed ideal agreement between the prediction and real observations, and the nomogram decision curve analysis performed well. The nomogram model can provide an accurate prediction of severe abdominal pain in patients with HCC after first cTACE, aiding in the personalization of pain management and providing novel insights into hospital nursing.

## Introduction

Hepatocellular carcinoma (HCC) is the sixth most common cancer and third leading cause of cancer-related deaths worldwide. Approximately half of all patients with HCC are Chinese^1^. Hepatectomy, ablation, and liver transplantation are potentially curative treatments. However, more than half of all patients with HCC are diagnosed at an advanced stage and, thus, unable to undergo surgery^2–4^. The therapeutic effect of transarterial chemoembolisation (TACE) has been evaluated in randomized trials and meta-analyses^5–7^, and it has been widely recommended as a standard treatment for these patients^8^. TACE types include conventional TACE (cTACE) and drug-eluting beads TACE.

However, adverse events after TACE have a prevalence of 87%, including nausea, vomiting, fever, abdominal pain, liver abscess, gallbladder gangrene and hepatic failure, etc.^9^. Among them, abdominal pain has the most serious effect on the quality of life of patients, causing anxiety. Previous studies have focused on post-embolisation syndrome (PES); some possible influencing factors were analysed, and relevant prediction models were established. To our knowledge, no study specifically focusing on severe abdominal pain after initial cTACE therapy for HCC has been conducted^10^.

This study aimed to evaluate the frequency of severe abdominal pain after first cTACE in patients with HCC, as well as explore the relevant risk factors. This data was used to establish a nomogram to predict severe abdominal pain after first cTACE, thereby improving pain management and providing individualized treatment and a model to establish post-cTACE care protocols for patients with HCC.

## Methods

### Study design and patients

In this retrospective study, all patients with HCC were diagnosed with HCC between the 28th of October, 2019, and the 5th of August, 2022, and treated with cTACE in the affiliated hospital of the Guilin Medical University.

HCC was diagnosed by non-invasive methods as the European Association for the Study of the Liver and the American Association for the Study of Liver Disease guidelines. The inclusion criteria were as follows: (1) age > 18 years, (2) an Eastern Cooperative Oncology Group performance status of 0 to 1, and (3) TACE is indicated for those patients who belong to BCLC-B are not suit for radical resection or ablation. For BCLC-A patients, TACE is recommended if patients are unable or unwilling to receive surgery or ablation for other reasons such as older age and severe cirrhosis. For BCLC-C patients, TACE is recommended as an option if patients with incomplete obstruction of the main portal vein or formation of abundant compensatory collateral branches of the portal vein^11^. (4) No treatment prior to cTACE, including local and systemic therapy. The exclusion criteria were as follows: (1) coexistence with other primary malignant diseases; (2) severe abdominal pain before cTACE; (3) concomitance of tumour rupture; (4) extrahepatic metastasis; and (5) insufficient clinical and baseline data.

The protocol was performed in accordance with the guidelines outlined in the Declaration of Helsinki and was approved by the Ethics Committee of Affiliated Hospital of Guilin Medical University. Since the study was a retrospective study, most of the study subjects have died or lost contacts, and all statistics were anonymous, so the Ethics Committee of Affiliated Hospital of Guilin Medical University agreed to waive the need for informed consent.

### TACE procedure

All the patients underwent initial cTACE. Half an hour before cTACE, all patients were routinely administered pethidine hydrochloride. A microcatheter was inserted through the segmental or subsegmental supply arteries. Chemoembolisation mixed 20–60 mg pirarubicin, 200 mg oxaliplatin, 5–20 mL lipiodol, and gelatine sponge. The injection volume of the emulsion depended on tumour volume. All cTACE procedures were performed by interventional radiologists who had over 6 years of experience.

### Pain assessment

Pain was assessed within 24 h after cTACE by using the visual analogue scale (VAS) score, which is a standard and checked ten-point scale for the self-reporting of pain^12^. Scores range from 0 to 10, with a score of 0 representing absence of pain and a score of 10 representing the highest pain level. Score between 1 and 3, 4 and 6, and 7 and 10 represented mild, moderate, and severe pain, respectively. When the pain level assessment is complete, we used acetaminophen, aminotriol ketorolate, and pethidine hydrochloride to relieve mild, moderate and severe pain respectively and immediately.

### Clinical data collection

The following clinical characteristics were collected: sex, age, maximum tumour diameter or tumour size, portal vein thrombosis, duration of the procedure, white blood cell count, red blood cell count, haemoglobin, platelet count, α-fetoprotein, alanine transferase, and aspartate aminotransferase. Access to the hospital’s medical record management system was used to obtain all data. Two investigators entered data simultaneously to ensure the accuracy of information. Two radiologists with a minimum of 5 years of experience independently reviewed all radiology images. If there were more than two tumours, patients were deemed to have multiple-lesion HCC, and the largest tumour, as indicated by diameter, was analysed.

### Statistical analysis

IBM SPSS version 27.0 (SPSS Inc., Chicago, IL, USA) was used for statistical analysis. Baseline characteristics were summarised using descriptive statistics. The Mann–Whitney *U* test was used to compare continuous variables, and either Pearson's Chi-squared test or Fisher's exact test was performed to compare categorical data. Quantitative data are expressed as frequency, mean ± standard deviation, or median with 95% confidence interval (CI) values. The statistical significance of the clinical factors was assessed by univariate analysis, and variables found to have statistical significance were included in the multivariate analysis using binary logistic regression to identify the risk factors associated with severe abdominal pain following cTACE.

A predictive nomogram was generated based on several independent factors evaluated by multivariate analysis using the R software version 3.2.0 (The R Foundation for Statistical Computing, Vienna, Austria) and the ‘rms’ package. Moreover, the model was validated using 1000 bootstraps to quantify the overarching modelling strategy while assessing the model's prediction accuracy. Each statistical test in our study was two-sided, and P-values < 0.05 were used to indicate statistical significance.

## Results

### Baseline characteristics

A total of 216 patients who underwent cTACE therapy were included in this analysis. Of these patients, 127 (58.8%) complained of severe abdominal pain after their initial cTACE. Of all patients who underwent cTACE therapy, 181 were men, and 35 were women. Most patients were younger than 65 years and had a tumour exceeding 5 cm in diameter. Portal vein tumour thrombi were present in 76 (35.1%) patients. The number of patients with α-fetoprotein levels above or below 400 ng/mL did not differ significantly between both groups. Additional details on patient characteristics are presented in Table 1.

**Table 1:** Baseline demographic and clinical characteristics of patients in the training and validation cohorts (n = 216).

Characteristics	Total (n = 216)	Training cohort (n = 151)	Validation cohort (n = 65)	P-value
Severe pain				0.376
Yes	133 (61.57)	91 (60.26)	42 (64.62)	
No	83 (38.43)	60 (39.74)	23 (35.38)	
Sex, n (%)				0.078
Female	34 (15.74)	19 (12.58)	15 (23.08)	
Male	182 (64.23)	132 (87.42)	50 (76.92)	
Age, n (%)				0.658
< 65 years	157 (72.69)	109 (72.19)	48 (73.85)	
≥ 65 years	59 (27.31)	42 (27.81)	17 (26.15)	
Hepatitis B, n (%)				
Yes	201 (93.10)	140 (92.72)	61 (93.85)	
No	15 (6.90)	11 (7.28)	4 (6.15)	0.432
Tumour location				0.219
Left lobe	17 (7.87)	10 (6.62)	7 (10.77)	
Right lobe	136 (62.96)	96 (63.58)	40 (61.54)	
Two lobes	63 (29.17)	45 (29.80)	18 (27.69)	0.367
Tumour size, n (%)				0.873
≤ 5	58 (26.85)	40 (26.49)	18 (27.69)	
> 5	158 (73.15)	111 (73.51)	47 (72.31)	
Tumour number, n (%)				0.097
Single	89 (41.20)	56 (37.09)	33 (50.77)	
Multiple	127 (58.80)	95 (62.91)	32 (49.23)	
PVTT, n (%)				0.440
No	141 (65.28)	101 (66.89)	40 (61.54)	
Yes	75 (34.72)	50 (33.11)	25 (38.46)	
Major vascular invasion				
Yes	8 (3.70)	5 (2.31)	3 (4.62)	
No	208 (96.30)	146 (97.69)	62 (95.38)	0.532
Dosage of lipiodol				0.387
< 10 mL	96 (44.44)	71 (47.02)	25 (38.46)	
≥ 10 mL	120 (55.56)	80 (52.98)	40 (61.54)	
Child–Pugh				0.548
A	157 (72.68)	109 (72.19)	48 (73.85)	
B	59 (27.32)	42 (27.81)	17 (26.15)	
Procedure time, mins	78.00 (64.00, 97.00)	77.00 (64.25, 97.75)	78.00 (64.00, 96.50)	0.647
WBC, 10^9^	6.30 (4.90, 7.72)	6.35 (4.89, 7.66)	6.30 (4.96, 7.78)	0.962
RBC, 10^12^	4.61 (4.09, 5.10)	4.66 (4.16, 5.14)	4.46 (3.97, 4.98)	0.414
Hb, g/L	138.00 (123.00, 152.00)	139.50 (125.00, 152.00)	133.00 (121.00, 147.50)	0.261
PLT, 10^9^	196.00 (134.00, 257.00)	191.50 (130.50, 257.00)	199.00 (149.50, 258.00)	0.706
AFP, n (%)				0.628
≤ 400 ng/mL	113 (52.31)	77 (50.99)	36 (55.38)	
> 400 ng/mL	103 (47.69)	74 (49.01)	29 (44.62)	
TBIL, nmol/L	13.8 (10.81, 17.45)	14.2 (10.80, 16.94)	13.5 (10.94, 17.85)	0.253
DBIL, nmol/L	5.5 (4.24, 6.86)	5.6 (4.03, 6.77)	5.4 (4.33, 7.01)	0.368
Albumin, g/L				0.487
< 35	15 (6.94)	11 (7.28)	4 (6.15)	
≥ 35	201 (93.06)	140 (92.79)	61 (93.85)	
ALT, U/L	45.00 (25.50, 68.00)	40.50 (25.58, 64.25)	55.00 (26.00, 69.00)	0.320
AST, U/L	54.00 (36.40, 97.00)	54.15 (36.25, 93.50)	51.00 (36.80, 108.50)	0.845

*PVTT* portal vein tumour thrombus, *WBC* white blood cell count, *RBC* red blood cell count, *Hb* haemoglobin, *PLT* platelet, *AFP* α-fetoprotein, *ALT* alanine transaminase, *AST* aspartate transaminase.

### Factors influencing severe abdominal pain after initial cTACE therapy

The variables associated with severe abdominal pain after univariate analysis were age > 65 years (P = 0.004), presence of multiple tumours (P = 0.001), tumour diameter > 5 cm (P = 0.001), and procedure time (P = 0.034). Multivariate binary regression analysis showed that age (P = 0.029), tumour size (P = 0.008), and tumour number (P = 0.019) were independent risk factors for severe abdominal pain (Table 2).

**Table 2:** Univariate and multivariate regression for severe abdominal pain after initial conventional transarterial chemoembolisation in the training cohort. Significant values are in bold.

Variables	Univariate		Multivariate	
	OR (95% CI)	*P*	OR (95% CI)	P-value
Sex				
Female	1			
Male	1.323 (0.446–3.929)	0.614		
Age				
< 65 years	1		1	
≥ 65 years	0.293 (0.128–0.672)	**0.004**	0.365 (0.148–0.904)	**0.029**
Hepatitis B				
Yes	1			
No	1.236 (0.763–3.652)	0.368		
Tumour location				
Left lobe	1			
Right lobe	1.339 (0.687–4.635)	0.463		
Two lobes	1.023 (0.879–3.265)	0.635		
Tumour size				
≤ 5 cm	1		1	
> 5 cm	5.735 (2.329–14.123)	**< 0.001**	3.702 (1.407–9.739)	**0.008**
Tumour number				
Single	1		1	
Multiple	4.008 (1.820–8.830)	**0.001**	2.822 (1.190–6.695)	**0.019**
PVTT				
No	1			
Yes	1.591 (0.715–3.541)	0.255		
Major vascular invasion				
Yes	1			
No	1.038 (0.637–3.245)	0.331		
Dosage of lipiodol				
< 10 mL	1			
≥ 10 mL	1.698 (0.535–4.298)	0.269		
Child–Pugh				
A	1			
B	2.036 (1.056–4.365)	0.364		
Procedure time	1.016 (1.001–1.032)	0.034	1.135 (0.934–1.332)	0.367
WBC	1.003 (0.877–1.149)	0.960		
RBC	1.001 (0.685–1.463)	0.995		
Hb	1.003 (0.989–1.018)	0.666		
PLT	1.002 (0.998–1.006)	0.266		
AFP				
≤ 400 ng/mL	1			
> 400 ng/mL	1.920 (0.910–4.050)	0.087		
TBIL, nmol/L	2.837 (1.026–6.235)	0.548		
DBIL, nmol/L	3.215 (0.968–5.897)	0.318		
Albumin				
< 35 g/L	1			
≥ 35 g/L	0.879 (0.687–3.914)	0.612		
ALT	0.997 (0.992–1.003)	0.324		
AST	0.998 (0.994–1.003)	0.495		

*OR* odds ratio, *CI* confidence interval, *PVTT* portal vein tumour thrombus, *WBC* white blood cell count, *RBC* red blood cell count, *Hb* haemoglobin, *PLT* platelet, *AFP* α-fetoprotein, *ALT* alanine transaminase, *AST* aspartate transaminase.

**Figure 1:**
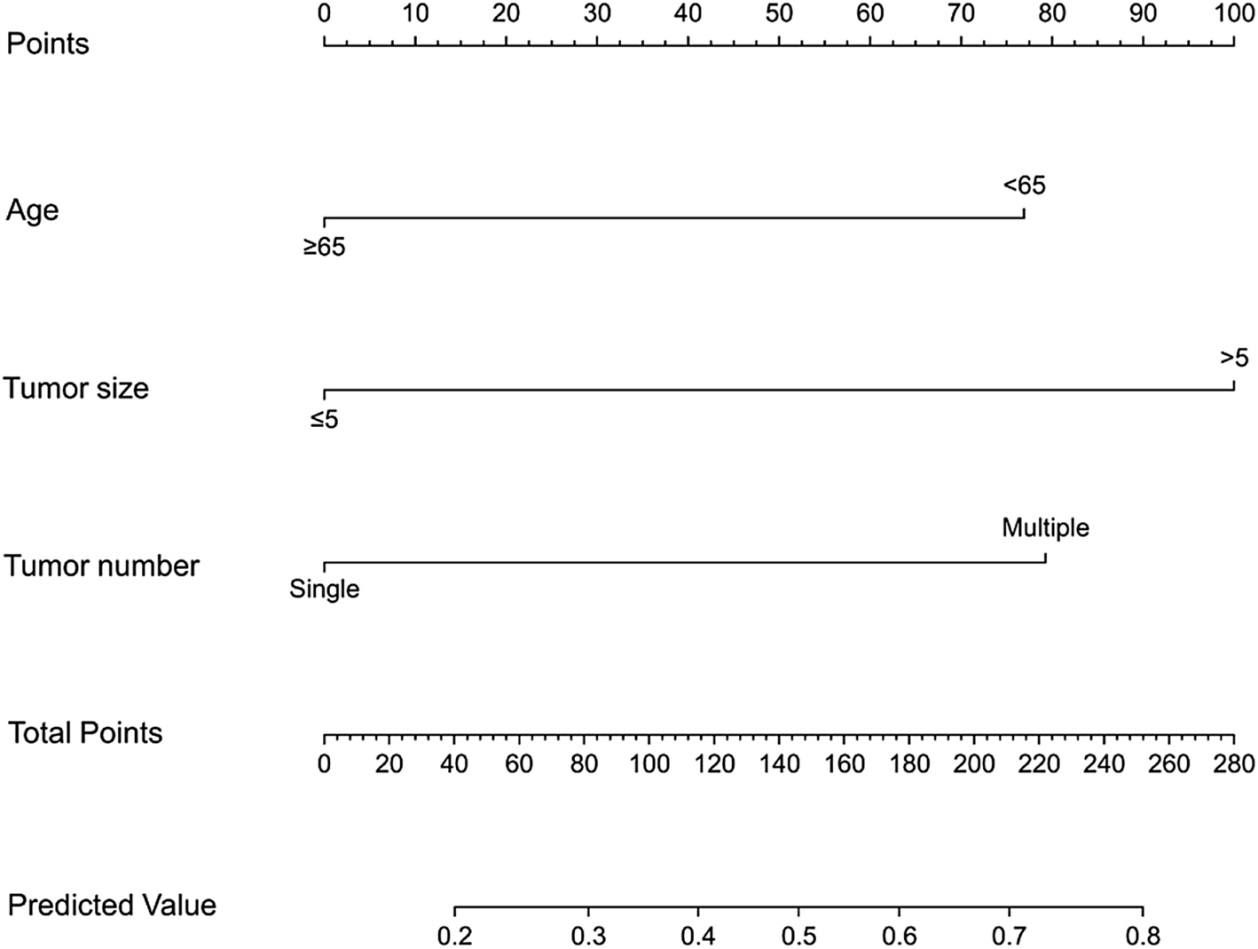
Nomogram to predict severe abdominal pain after initial conventional transarterial chemoembolisation in the training set.

Subsequently, we established a nomogram based on the significant risk factors identified by the univariate and multivariate analyses in the training set (Fig. 1). For each factor in the nomogram, a weighted number of points was calculated, and the sum of points for each patient was associated with a corresponding prediction of the presence of severe abdominal pain. A higher total score was associated with a higher rate of severe abdominal pain. For example, a 71-year-old man with a single 75-mm HCC nodule would have a total of 100 points (age, 0 points; tumour size, 100 points; and tumour number, 0 points). For this patient, the predicted incidence of severe abdominal pain was 38%.

### Model performance and validation

**Figure 2:**
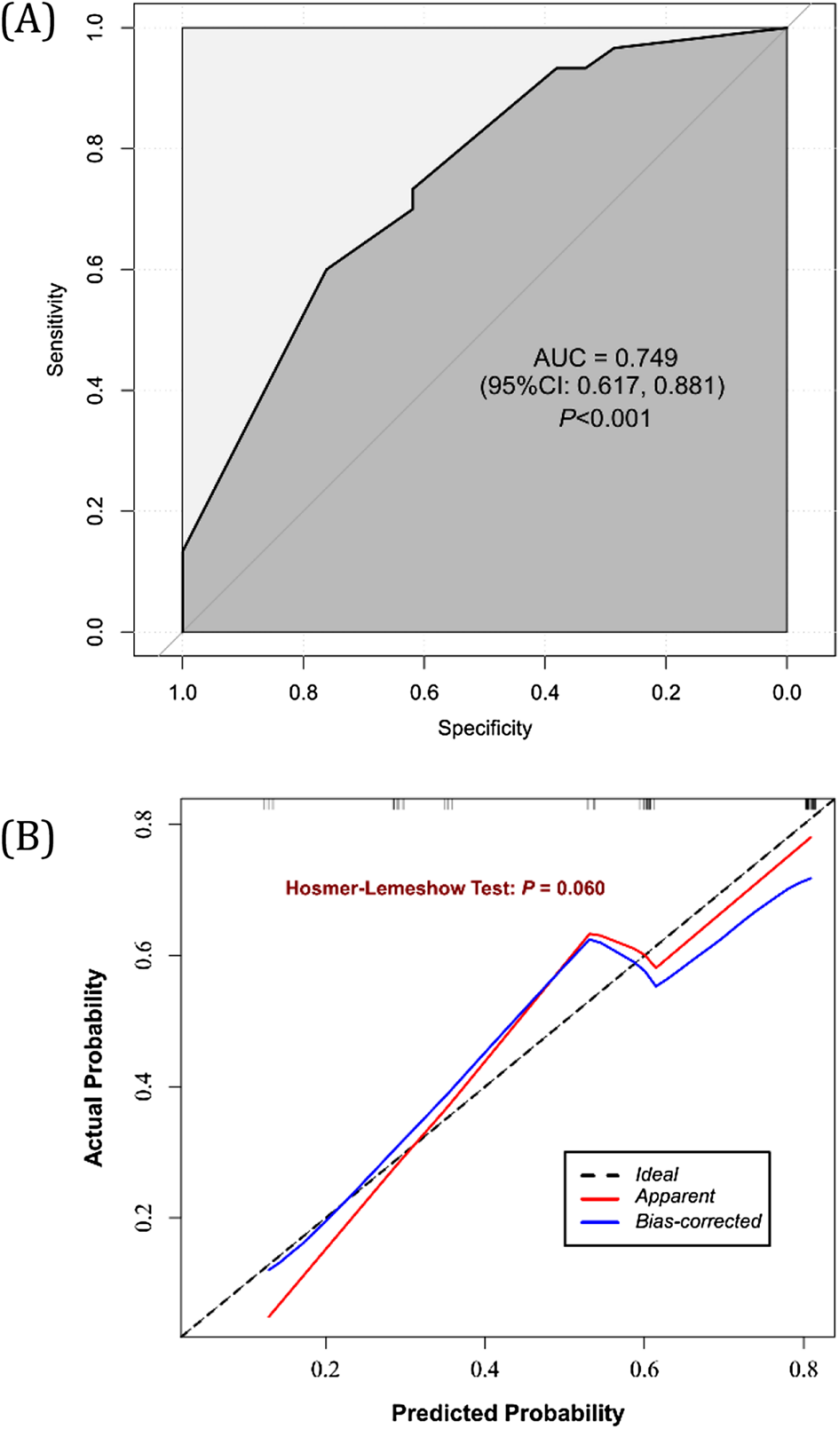
Receiver operating characteristic (**A**) and calibration curves (**B**) in the training set.

**Figure 3:**
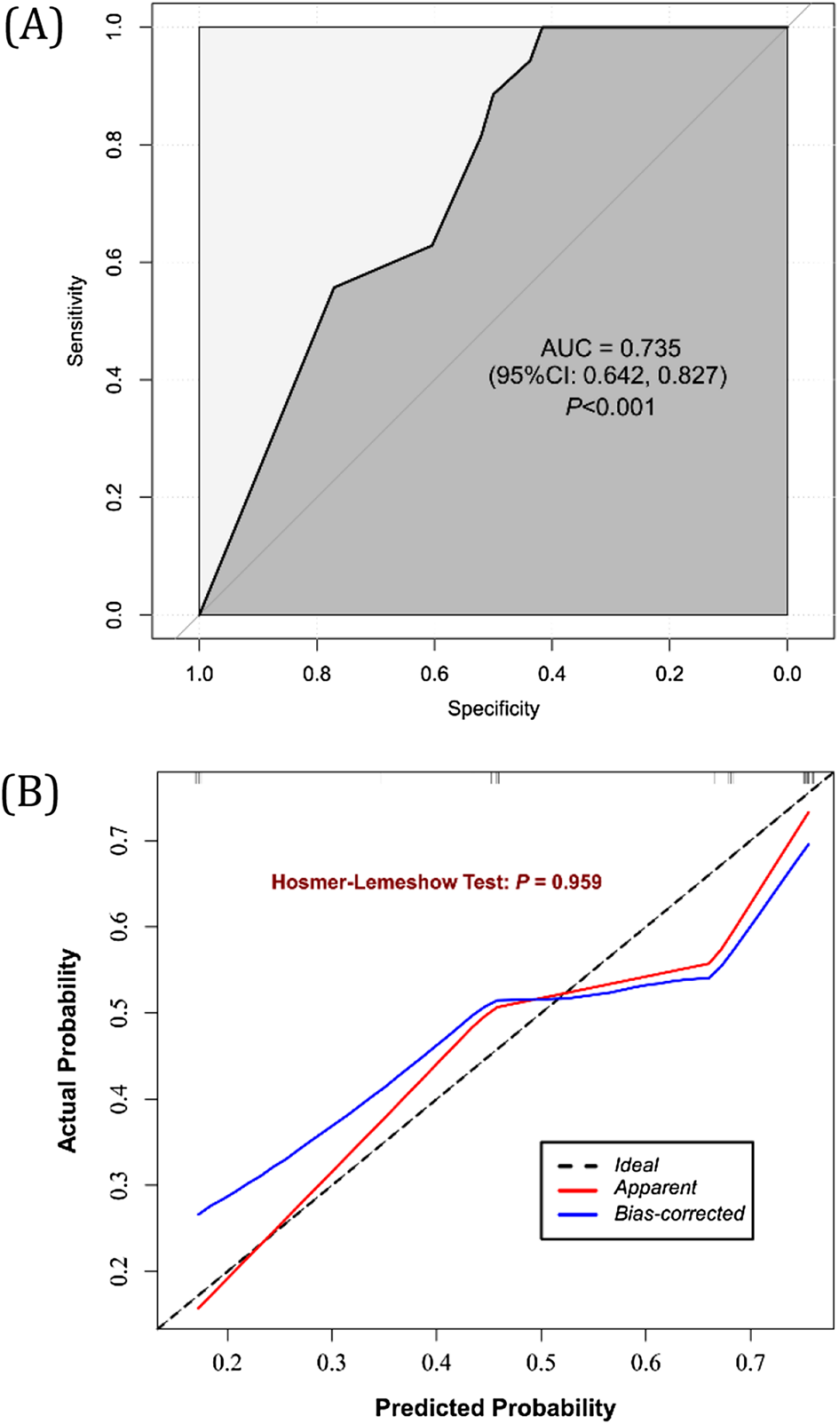
Receiver operating characteristic (**A**) and calibration curves (**B**) in the validation set.

**Figure 4:**
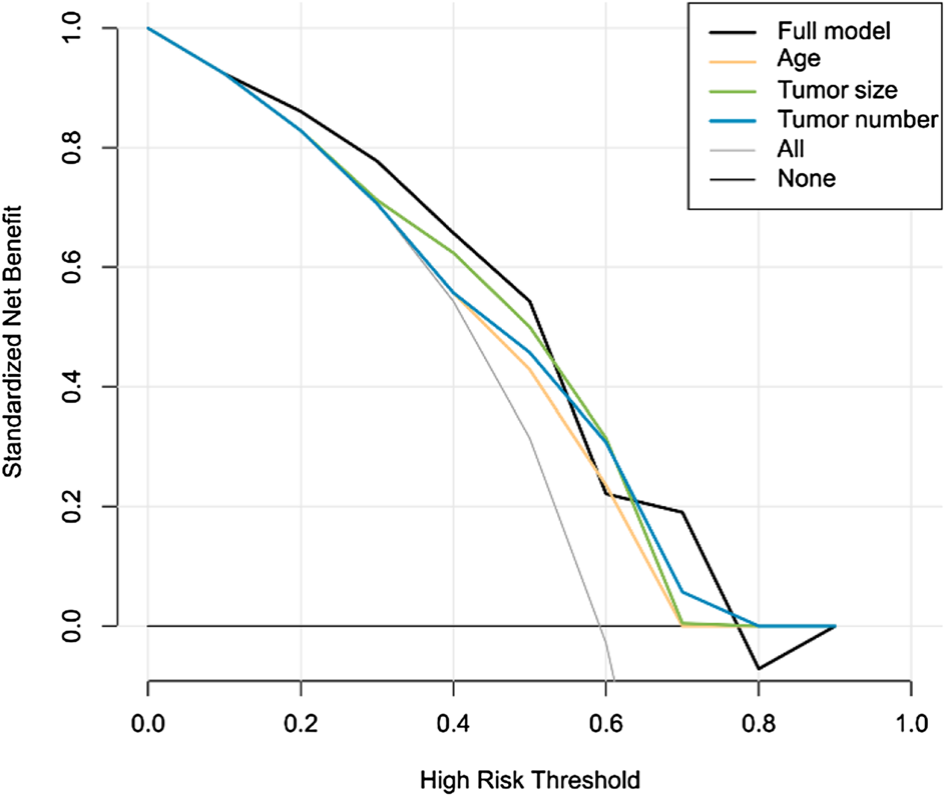
Decision curve analysis for severe abdominal pain in the training set.

The C-index of the nomogram within the training set was 0.749 (95% CI 0.617, 0.881). As seen in Fig. 2, the calibration curves in the training set demonstrated an optimal relationship. Furthermore, the calibration curves showed favourable calibration of the nomogram in the testing set (Fig. 3). The decision curve analysis (DCA) demonstrated that this nomogram had an added benefit in predicting severe abdominal pain as compared with treating all patients or treating no patient in the training and testing cohorts (Fig. 4).

## Discussion

Although surgical resection and liver transplantation may cure HCC, but most of HCC patients are already in the middle and advanced stages at the time of diagnosis, and they are not suitable for radical resection. Systemic therapy, including tyrosine kinase inhibitors (TKIs) and immune checkpoint inhibitors (ICIs), improved the prognosis for these patients in recent years. TACE is also an effective therapy and widely recommended as first-line therapy for intermediate and advanced HCC. However, the rate of adverse events after TACE is high. In our study, the probability of severe abdominal pain following the first cTACE procedure was as high as 58.8%, which is greater than the likelihood reported in other studies^13^. One possible reason for this is that our study included a relatively high number of younger patients than other studies. Bian et al. included other patients, including those undergoing their first TACE procedure, because a history of TACE procedure has been reported as a protective factor for post-embolisation abdominal pain^13^. The mechanisms underlying post-embolisation pain are still not completely understood. However, it is believed to be related to local tissue hypoxia, tumour necrosis, ectopic embolisation, and inflammatory response caused by cytokine release^14,15^.

Currently, few studies have explored the risk factors influencing the development of PES in patients with HCC after He et al. discovered that drug-loaded microsphere TACE and serum albumin could act as protective factors influencing PES, whereas drug loading was a risk factor for PES after the first TACE for patients with HCC^16^. Khalaf et al. illustrated the history of PES, tumour burden, and drug-eluting embolic TACE to be predictors affecting PES and, thus, constructed a predictive model for PES after TACE^17^. Moreover, Pachev et al. used VAS to identify factors predicting severe abdominal pain during and after TACE for HCC and found that age, liver cirrhosis, and alcoholic liver disease were negative predictive factors of severe abdominal pain^18^. However, to our knowledge, this study is the first to focus on the factors influencing severe post-embolisation abdominal pain after initial cTACE among patients with HCC and to establish a simple, easily applicable nomogram based on clinical characteristics. The nomogram displayed good discrimination power, with C-indices of 0.749 (95% CI 0.617, 0.881) in the training cohort and 0.728 (95% CI 0.592, 0.864) in the validation cohort. The calibration curves showed ideal agreement between the prediction and real observations. Finally, the DCA of nomograms performed well.

In our study, age, tumour number, and tumour size were independent predictors of severe abdominal pain, and thus construct the nomogram. These results provide clinically relevant information to help predict and proactively pain treatment, relieve patients' pain and therefore shorten their hospital stay.

According to the nomogram, the older the patient, the less severe the pain, which is consistent with the results of Pachev et al.^18^. The relationship between age and pain is complex and depends on the clinical situation. The most possible reason for this is that pressure nociception thresholds decrease with age^19^. Moreover, the tumour size and tumour number are referred to as tumour burden^20^. Our study found that higher tumour burden was indicative of more severe abdominal pain and was similar to that of Bian et al. in that patients with larger tumour sizes or multiple tumours required a longer procedure duration^13^. This is associated with drug dose and causes longer arterial spasms and more extended liver parenchymal necrosis, especially those of the bile ducts. Bile ducts are considered sensitive to oxygen deprivation^14,21^.

cTACE treatment can cause various PES besides abdominal pain, including nausea, vomiting, chill, fever, liver infarction, liver abscess, heterotopic embolization, acute renal failure, acute hepatic failure, and so on. For the prevention of these complications, the dose of lipiodol should be well monitored and superselective chemoembolization is required^22,23^. Many previous studies have illustrated the risk factors of other TACE complications, such as acute hepatic failure or nausea and vomiting and have shown that liver enzymes is one of the main predictive risk factors^24,25^. But in our study, liver function (bilirubin, albumin, ALT, AST) levels were not associated with severe abdominal pain.

Our study has notable limitations. First, the sample size was relatively small, and the retrospective analysis has intrinsic limitations. Our nomogram model should, therefore, be verified by conducting a large prospective study and including other potential contributory factors. Moreover, most patients included in this study had an aetiology of hepatitis B virus infection. Thus, our nomogram model should be verified by including other HCC aetiologies. Furthermore, our model was not externally validated. Future studies with a larger sample size across multiple sites should be designed to confirm our findings. At last, our prediction model included only one complication, severe abdominal pain, and we will include others in future study.

## Conclusions

In conclusion, we generated a nomogram to predict severe abdominal pain after cTACE in patients with HCC. A reliable and feasible non-invasive approach to predict severe post-embolisation abdominal pain in patients with HCC may improve treatment in clinical practice and provide personalized treatment.

## Data Availability

The data used in this study can be obtained from the corresponding author on reasonable request.

## Abbreviations

HCC: Hepatocellular carcinoma
TACE: Transarterial chemoembolization
cTACE: Conventional TACE
PES: Post-embolisation syndrome
VAS: Visual analogue scale
CI: Confidence interval
DCA: Decision curve analysis
